# Hygromorphic Response Dynamics of 3D-Printed Wood-PLA Composite Bilayer Actuators

**DOI:** 10.3390/polym13193209

**Published:** 2021-09-22

**Authors:** Daša Krapež Tomec, Aleš Straže, Andreas Haider, Mirko Kariž

**Affiliations:** 1 Biotechnical Faculty, University of Ljubljana, Jamnikarjeva 101, 1000 Ljubljana, Slovenia; dasa.krapez.tomec@bf.uni-lj.si (D.K.T.); ales.straze@bf.uni-lj.si (A.S.); 2 Kompetenzzentrum Holz GmbH, 4040 Linz, Austria; a.haider@wood-kplus.at

**Keywords:** 3D printing, 4D printing, actuators, wood, dimensional changes, shape-changing materials

## Abstract

The use of wood particles in wood-plastic composites (WPC) is well known and similar use could occur in materials for fused deposition modeling (FDM) 3D printing. Wood particles could be one of the possible solutions in the search for natural-based materials to minimize the use of synthetic-origin materials in additive manufacturing. Wood particles for 3D printing filaments can be made from wood waste and could serve as a cheap filler or as a value-added reinforcing component, depending on their properties and incorporation. The disadvantages of wood (dimensional changes due to water adsorption and desorption) could be used as functions when dimensional change is desirable, such as in shape-changing 4D printing materials. In this research, FDM printing materials made of polylactic acid (PLA), with different amounts of wood particles, were used to design moisture-induced shape-changing bilayer actuators, which could serve as a principle for active façade or ventilation valves. The initial research shows that the wood content in the WPC causes dimensional changes and thus shape changes of the designed actuators under changing climates. The shape change depends on the ratio of the materials in the two-layered actuator and the wood content in the wood-PLA composite used, and thus on sorption. The rate of the shape change behaves in the same way: the higher the wood content, the greater the change observed. The dynamics of the hygromorphism of bimaterial composites is greater with a small amount of added hygromechanically active material.

## 1. Introduction

The use of natural fibers and particles in wood-plastic composites has been increasing in recent decades, including in materials for 3D printing [1]. Natural fibers/particles are used as reinforcement in polymer composites to replace synthetic fibers due to their mechanical, acoustic, or even morphing properties [2] in combination with their low density, reduced environmental footprint, and alternative end-of-life management [3,4]. As a natural fiber composite material, wood has various complex properties that allow it to compete with modern high-tech materials. However, processing particles for use in FDM 3D-printed polymer composites can be time- and energy-consuming, requiring milling, grinding, or even chemical degradation to a sufficient size [1].

Natural fibers are anisotropic and moisture sensitive, which is one of their main disadvantages when used for structural applications [3], but could also be used as a positive attribute when shape change is desired, as in 4D printing or when used as shape-memory materials/stimuli-responsive materials. 4D printing has evolved from 3D printing and aims to achieve a predictable and predefined time-dependent change in functionality (shape, property, self-assembly, or self-repair) that the 3D-printed structure undergoes when it encounters an external stimulus (e.g., temperature, ultraviolet light, humidity, electric and magnetic fields) [5]. Actuation in response to a stimulus by pre-programmed hierarchical structures can provide a bio-inspired model useful for the functional gradation of natural fibers to develop hygroscopically induced morphing (i.e., hygromorphing). The difference in volumetric expansion, bending stiffness, and modulus of elasticity of each layer forms the basis for responsive deformation behavior [6]. In 3D-printed structures, these properties can be influenced by proper design of the model, selection of the material, and printing properties (e.g., wall thickness, infill density, infill pattern, printing line orientation, etc.).

Many variables characterize the strength of parts made with FDM, so it is difficult to determine those that most affect structure-property correlations. The influencing variables may also be material-dependent and interrelated, so further research in this direction is essential [1,7,8]. Porosity due to infill patterns and infill percentage, as well as the voids caused by the extrusion process (voids between the deposition lines) and voids within the filament (detected by 3D computed tomography) affect the mechanical properties [9,10,11,12]. The mechanical properties of a 3D-printed element are reduced with poor interfacial bonding between the more hydrophobic PLA and the hydrophilic natural fiber and are decreased with greater layer height [13] and lower infill and density, depending on the layer orientation.

When using fibers in printing materials, the properties of 3D- and 4D-printed composite materials are additionally affected by the type of fibers used as well as their stiffness, strength, and capacity for interfacial bonding with the polymer matrix. In an ideal scenario, the microstructure of the composite material would be homogeneous, with well-distributed fibers with a high aspect ratio (length/diameter), and the interfacial surface would be maximized to ensure load transfer. To meet this goal, defects or porosity in the matrix and interfacial area should be reduced as much as possible [14]. The anisotropic swelling of natural fibers has been used as a driver of actuation in the development of hygromorphic biocomposites via 3D printing. Fiber content, fiber orientation control, and fiber continuity are outlined in relation to known actuation performance challenges [14].

The principle of hygroscopic actuation is well known and present in nature (e.g., pinecones). It is based on a hierarchical, two-layered microstructure consisting of sclerenchyma and sclereids. Each of these tissues is organized as a bundle of individual fibers, with each fiber being a concentric compound cylinder composed of different cell walls. The secondary cell wall, which is mainly responsible for the hygromechanical properties of the single fiber, consists of oriented stiff cellulose microfibrils embedded in the hygroscopic hemicellulose/pectin matrix. The bilayer structure results in differential swelling between two layers connected by a gradual interphase [14].

Hygromorphic or shape-changing materials work differently, because it is the external stimulus that triggers the transformation from their original shape; the transformation is reversed when the stimulus is removed. Therefore, the material can be considered to oscillate between two equilibrium states, without the need for an external force, allowing for multiple cycles of transformation. However, the direction and amplitude of the motion are pre-programmed into the material structure [15].

Two subsets of architectural applications are of particular interest to this discussion: opening roofs for semi-interiors, such as sports stadiums, and adaptive facades for fully enclosed buildings. Adaptive facades attempt to achieve responsiveness through more localized permeability control, while most convertible roofs utilize the movement of larger building components. Both systems typically have a high degree of mechanical complexity and require at least one external power source, actuators and sensors, and a logical control unit [16]. The instrumentalization of hygroscopic material behavior is particularly promising for architecture because it does not require any kind of external actuation, electrical or otherwise.

In contrast to manipulating the natural wood grain, 3D printing technologies enable designing specific wood grain patterns to precisely control the direction of curling. In addition, by using multi-material printing, with different materials in combination with wood, custom wood composites that produce new macro-level behavior can be created. These composites take advantage of the natural expansion and contraction properties of wood, enhance its transformation properties, and provide greater control over the desired curvature [17].

At the microscopic level, fiber orientation dictates the direction of expansion, and within the polymer composite, fibers are primarily oriented during filament production rather than during printing [18]. However, the printing trajectories and the orientation of the screen pattern create oriented mesostructures in the printed structure, which are important for promoting complex actuation. Correa and co-authors [17] also suggest that a certain amount of shear-induced alignment in the nozzle may arrange some fibers longitudinally along the extrusion path.

The addition of wood to PLA creates a hygromechanically active composite material that can deform as moisture is adsorbed/desorbed in changing climates. The aim of this research was to design bimaterial actuators of PLA and wood-PLA composites with different percentages of wood particles, and to investigate the moisture sorption and transport in relation to the mechanical properties and structure of these composites when exposed to a climate that can change. A new contribution of this study is to explain the less explored area of wetting dynamics with the prevailing internal resistance to moisture transfer on the hygromorphic bending of these composites.

## 2. Materials and Methods

Three different filaments were used for 3D printing: one commercially available PLA (Plastika Trček, Slovenia) and two wood-PLA filaments; one with 15% (WPL15) and one with 25% wood content (WPL25), manufactured by Kompetenzzentrum Holz GmbH (Linz, Austria). For production of these two wood-PLA filaments, PLA type Ingeo 3251 D from NatureWorks LLC and wood flour with particle size 70 to 150 μm (Arbocell C100 supplied by Rettenmaier & Söhne GmbH&CoKG, Rosenberg, Germany) were used. The production parameters, 3D computed tomography, and microscopic images of the wood-PLA of the two aforementioned filaments are available in previous studies by Ecker and co-authors [12] and Kain et al. [8].

Wood particles (flour) were used because of the possibility of more direct use of wood residues, lower price, less energy and labor required for material preparation, and the fact that no chemicals are used for production.

All samples were printed using a Creality CR10-V3 3D printer (Creality 3D Technology Co., Ltd., Shenzhen, China) with a direct extruder. The print layer thickness was set to 0.3 mm, the nozzle diameter to 0.4 mm, the printing temperature to 200 °C, and the bed temperature to 50 °C. The samples were printed with solid layers, with printing lines at an alternating angle of 45° (one layer +45°, next layer +45°) to the sample length.

The investigations were carried out in two steps: first, the hygroscopicity, dimensional stability, water vapor diffusivity, and bending stiffness of the printed samples were determined at a constant temperature and varying relative humidity. In this part, the samples were printed with only one material. In the second step, the behavior of the bimaterial actuators was determined; the samples were made of two materials: PLA and one of the wood-PLA composites.

### 2.1. Hygroscopicity and Dimensional Stability of the Source Polymers

Samples for the first step (i.e., dimensional stability—humidification/drying tests) were modeled in SolidWorks software (SolidWorks Corp., Waltham, MA, USA) and exported in the STL format. The STL models were sliced and prepared for 3D printing using Cura software (Ultimaker, Utrecht, Netherlands). The dimensions of these specimens were 120 mm × 15 mm × 4 mm (length × width × thickness).

The dimensional stability tests were performed in the laboratory drying tunnel TLS-01 (Kambič, Semič, Slovenia). In the test chamber of the drying tunnel, with dimensions 700 mm × 400 mm × 610 mm (length × height × width), a supporting grid floor was placed in the middle, on which three series of seven specimens were positioned. The humidification and drying processes were controlled using a DPC-420 central microprocessor controller (Kambič, Semič, Slovenia), which allowed the adjustment of air temperature (T), relative humidity (RH), and air velocity (v) (ΔT = ±1.0 °C, ΔRH = ±1.0%, Δv = ±0.1 m/s).

After the samples were 3D printed, they were conditioned in a climate chamber at 20% RH. To measure the adsorption and desorption kinetics, the samples (n = 7) were first exposed to 80% RH (humid climate) for seven days and then to 20% RH (dry climate) for the next seven days. The temperature was a constant 20 °C, and the air velocity was 1 m/s. The sorption process of the 3D-printed samples was monitored by interval weighing of each sample on an Exacta 300 EB laboratory balance (Tehtnica, Železniki, Slovenia) with an accuracy of 0.01 g and by manual measuring the three dimensions of the samples with a digital caliper with an accuracy of 0.01 mm.

### 2.2. Analysis of the Humidification and Drying Kinetics of the Source Polymers

The moisture content change and, consequently, the dimensional stability and shape change of 3D-printed source polymers can be seen as similar to solid wood studied in unsteady state conditions by the response of the system to an instantaneous, constant external perturbation. This is characterized by the transition of the system to a new steady state, which can be described as a first order system (FOS) with a differential equation and from which the response of the sample could be derived and determined by monitoring the change of mass and consequent expansion or contraction of the samples, and calculating the time constant (τ-tau) using Equation (1) [19,20]:

(1) $$G\Phi \left(t\right)=\tau \frac{\mathrm{dm}}{\mathrm{dt}}+m$$

G—stationary system response in g,
Φ (t)—transient system response,
τ—time constant in s,
M—mass in g,
t—time in s.

The initial mass of the sample (m_i_), the mass of the sample at a certain time (m_t_), and the final or equilibrium mass (m_f_) are introduced in the above equation to be equivalent to the stationary response (G), which is reached after a sufficiently long conditioning time. The above equation for the case of instantaneous loading at time t = 0, with the initial condition m = m_i_, can be then written in the following form:

(2) $$m_{t}=m_{f}+\left(m_{i}-m_{f}\right){\;*\;e}^{-\frac{t}{\tau }}$$

By transforming the expression (Equation (2)), we can obtain the dependence of the average dimensionless change in mass, i.e., moisture ratio (MR; Equation (3)):

(3) $$\mathrm{MR}=\frac{m_{t}-m_{f}}{m_{i}-m_{f}}=e^{-\frac{t}{\tau }}$$

The variable (m_t_) reaches 63.2% of the instantaneous load G when the condition **t** = **τ** is reached. The final response of the system (99.9%) is usually achieved after 5**τ** [19,20]. By monitoring the mass of the samples, we calculate the time constant by using a logarithm on the expression (Equation (4)):

(4) $$\tau =-\frac{t}{ln\left(\frac{m_{t}-m_{f}}{m_{i}-m_{f}}\right)}$$

### 2.3. Determination of Diffusion Coefficient of Moisture Transport

Fick’s theoretical model of mass transfer was used to analyze the kinetics of both the humidification and drying process. The model assumes that diffusivity is the sole physical mechanism responsible for the transfer of moisture to the wood surface. The relation between the moisture ratio (MR) and the effective diffusion coefficient is given by Crank [21]; this was used for slab geometry, assuming that the initial moisture concentration is uniformly distributed in the specimen. This relation was reduced to Equation (5), and it was assumed that the specimen is homogeneous, that mass transfer through the specimen is controlled by bound water, and that the water vapor diffusion and the effect of shrinkage is negligible.

(5) $$\mathrm{MR}=\frac{8}{\pi^{2}}\exp\left[\frac{-\pi^{2}\cdot D_{\mathrm{eff}}\cdot t}{4\cdot L^{2}}\right]=\frac{8}{\pi^{2}}\exp\left[\frac{-t}{\tau }\right]$$

where L is the half-thickness of the specimen (m), D_eff_ is the effective diffusion coefficient in m^2^/s, and t is the humidification/drying time in seconds. By plotting *ln (MR)* versus humidification/drying time, D_eff_ was determined from the slope (k) of the line 1/τ (Equation (6)).

(6) $$D_{\mathrm{eff}}=\frac{4L^{2}}{\pi^{2}\;}\frac{1}{\tau }$$

### 2.4. Bending Stiffness of Source Polymers

The modulus of elasticity (MOE) of the 3D-printed specimens of selected materials was determined on the same specimens used to determine dimensional stability (Section 2.1). MOE was determined after conditioning in four climates, with varying RH (20%, 40%, 65%, and 80%), at 20 °C for 168 h.

The specimens were tested in a three-point bending test on the Zwick Z005 universal testing machine (ZwickRoell GmbH, Ulm, Germany). The support span L_s_ was 80 mm, and the loading rate was 10 mm/min. The specimens were loaded to F_2_, approximately 50% of the maximum loading force (for each material, one specimen was loaded to failure), and MOE was determined accordingly:

(7) $$MOE=\frac{L_{s}^{3}}{4bt}\frac{\left(F_{2}-F_{1}\right)}{\left(U_{2}-U_{1}\right)}$$

L_S_ is the distance between the center point loading and the support,

b and t are width and thickness of the specimen, respectively,

(F_2_ − F_1_) is the increment of the load on the straight line of the load-deflection curve,

(U_2_ − U_1_) is the increment of deflection corresponding to (F_2_ − F_1_).

The values given for each material were calculated as the average values of six specimens for each RH climate.

### 2.5. Determination of the Shape Change of Bimaterial Actuators

**Figure 1:**
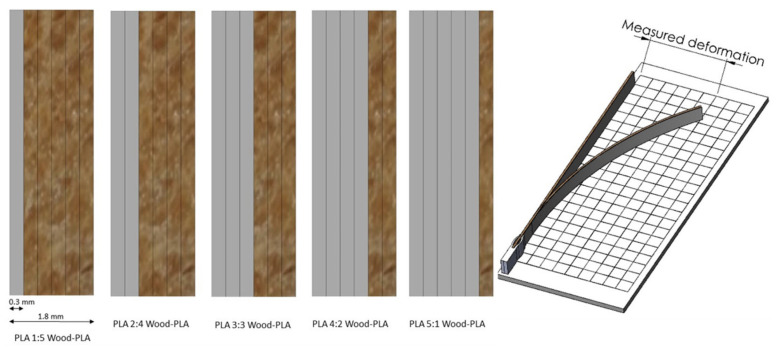
The composition of the actuator samples (**left**) and the mounted sample on the measuring template to determine the hygroscopic deflection (**right**).

The samples for the second step of research were made as bimaterial samples, consisting of six layers; the first layers (1–5 × 0.3 mm) were printed with pure PLA, and the other layers (5–1 × 0.3 mm) with wood PLA composite (either WPL15 or WPL25). 3D models with the dimensions 200 mm × 12 mm × 1.8 mm (length × width × thickness) were modelled in SolidWorks software (SolidWorks Corp., Massachusetts, MA, USA) and exported to STL format. The final thickness was always 1.8 mm, and the ratio between PLA and wood PLA layers varied (1:5, 2:4, 3:3, 4:2, 5:1) (Figure 1).

STL models were sliced and prepared for 3D printing in Cura software. Immediately after printing, the specimens were clamped on one end for approximately 10 mm on a specially designed template. First, they were conditioned in 20% RH and 20 °C for at least 72 h. Afterwards, the samples were weighed together with the template, and the initial position was measured and marked with a millimeter grid (Figure 1).

The samples were then placed in a humid thermostatic climate with 80% relative humidity at 20 °C. At predetermined time intervals, the deviation of the endpoint from the initial position of each sample was monitored and marked with dots on the measuring template, and, in parallel, the samples were weighed on an Exacta 300 EB laboratory balance with an accuracy of 0.01 g. After one week of humidification, the samples were returned to dry thermostatic climate (20% RH and 20 °C), and the measurement of deflections during drying was monitored in the same way.

The axial expansion or contraction process, i.e., swelling and shrinking in 3D-printed samples in a transient state, can be studied in the same way as the response of the system’s mass to an instantaneous, constant external perturbation (Equation (1)). By observing the deviation of the endpoint of the sample (L = √(Δx^2^ + Δy^2^)) from the initial state L_i_, through the transition process in discrete time intervals (**L_t_**) to the final state (**L_f_**), the time constant was calculated for the process of the hygromechanical bending of the samples (Equation (7)) similarly to the mass change of the system (Equation (4)). The hygromechanical bending of the bimaterial actuator is explained as a consequence of the different axial expansion/contraction of two combined materials, which also have different water vapor sorption properties.

(8) $$\tau =-\frac{t}{ln\left(\frac{L_{t}-L_{f}}{L_{i}-L_{f}}\right)}$$

## 3. Results and Discussion

### 3.1. Sorption Dynamics and Dimensional Stability of Source Polymers

The results show that the dimensional stability of 3D-printed parts depends on the material used (Table 1). The lowest longitudinal swelling was observed in samples made of PLA, being 0.09% when samples were exposed from dry to humid climates (between 20% and 80% RH). Specimens made from wood-PLA exhibited higher longitudinal expansion (0.3% and 0.47%). Wood-PLA with higher wood content (WPL 25) had higher expansion. These results were expected and have been reported in previous studies [22,23,24]. Moisture adsorption increases with wood content in composites because of the free hydroxyl groups (-OH) of hemicelluloses and amorphous cellulose within the cell wall of wood fibers [25]. Therefore, wood-plastic composites with higher wood content adsorb more moisture when exposed to higher air humidity (Table 1). However, the differential shrinkage/swelling (%/% of moisture change) is the same for all tested materials; the difference between the materials is only in the attained equilibrium moisture content; thus, the total expansion of wood plastic composites is higher (differential shrinkage/extension, multiplied by moisture change).

Certain biopolymers, including PLA, are said to have a notable hygroexpansion; however, as can be seen from these results, the values are considerably lower compared to wood-PLA. However, PLA as a printing material is still considered a hygroscopic material, and filament and printer manufacturers recommend storing in dry, vacuum-sealed containers with silica gel to avoid moisture adsorbance.

**Figure 2:**
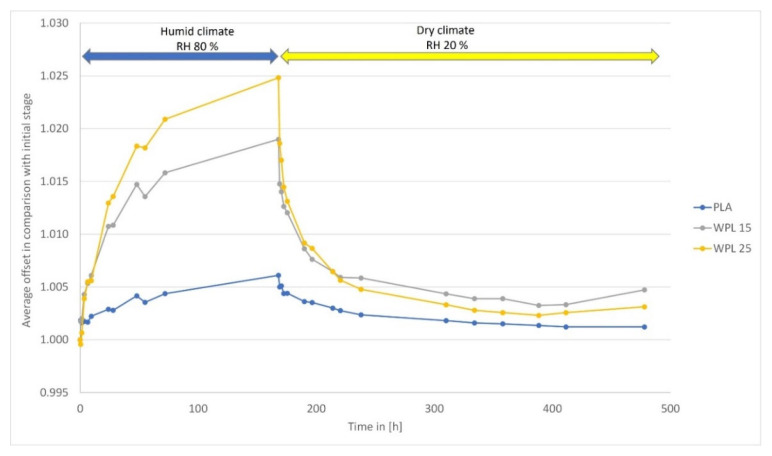
Adsorption and desorption curve of PLA, WPL 15, and WPL 25 specimens.

This study confirmed, similarly to previous research findings, that the adsorption rate and consequent axial expansion of polymers and biopolymers, in the case of building bimaterial composites, is temporally non-linear, with a rapid increase for a short time and a subsequent deceleration effect before reaching the saturation regime (Figure 2) [14]. Adsorption of moisture and moisture transport fare was found to obey FOS and the diffusion equations used (Equations (1)–(4)) [12,18].

The sorption hysteresis effect, where the equilibrium moisture content (EMC) achieved is higher in the case of desorption than adsorption, was also confirmed in this study (Figure 2). The causes of sorption hysteresis are the equilibrium time, energy losses due to plastic deformation, the formation of polar hydrogen bonds, the contact angle of capillary condensation, and internal stresses [26]. During desorption, the hydroxyl groups release water and saturate each other, while the cell wall shrinks. During subsequent adsorption, these hydroxyl groups do not bind the water immediately, which is also due to the compressive stress emanating from the structure of the adsorbate, resulting in a lower EMC during adsorption.

### 3.2. Moisture Diffusivity of Source Polymers

**Figure 3:**
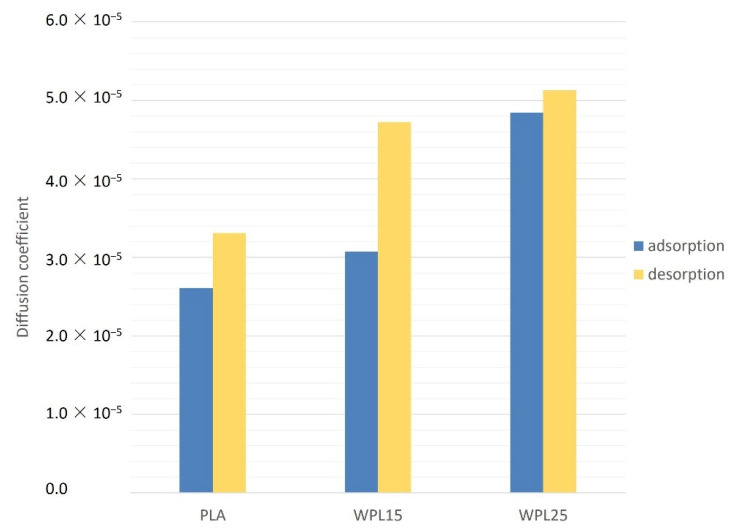
Diffusion coefficient at adsorption and desorption of PLA, WPL15, and WPL25 specimens.

Samples made from PLA have considerably lower diffusivity for moisture transport than samples made from wood-PLA, where the biopolymer with higher wood content (WPL25) had the highest diffusion coefficient (Figure 3). The diffusion coefficient was found to be higher, though not statistically significant (*t*-test; *p* > 0.05), during the desorption process. The overall diffusion of wood tissue is influenced by several factors, the most important of which are density, temperature, moisture content, flow direction, and fiber orientation [27]. Comstock [28] found that at a higher moisture content of wood, the diffusion coefficient is also higher, which might also be related to the higher diffusion coefficient in the case of WPL25, which, having higher wood content, gained more moisture during the humidification process (Table 1).

In wood-plastic composites, a comparable material to the 3D-printed WPL polymers of this study, in the case of higher content of wood particles, the moisture diffusion is mainly through wood particles; therefore, this WPC exhibits a higher diffusion coefficient. The diffusion coefficient in WPC increases with the content of wood particles and their size and decreases with the use of coupling agents, which increase the adhesion between wood and polymer [29]. Research also confirms that the sorption behavior of WPCs follows the kinetics and mechanisms described by Fick’s law, which was also used in the present study [29,30].

### 3.3. Stiffness in Bending of Source Polymers

The highest modulus of elasticity in bending was determined for the samples of pure PLA. The stiffness of the material with 25% wood content (PLA25) was higher than that of the material with a 15% wood share (WPL15) (Table 2). The results were expected and are consistent with previous reports [8,31,32,33]. Kariž and co-authors [24] identified small increases in MOE and tensile strength values measured at lower wood additions, but these decreased at higher loads. At low wood additions, the wood particles can act as reinforcement, but at higher loads, the polymer cannot fully encapsulate the particles, resulting in poor bonding and limited load transfer.

The mechanical properties of wood plastic composites also depend on the moisture content of the material. The highest stiffness of all tested materials was found after conditioning of the samples at 20 °C and 40% RH. Lower values were found in the driest climate (20 °C and 20% RH). The results might reflect the findings of solid wood, where the mechanical properties (including modulus of elasticity) tend to be lower below the MC of the saturation of the primary sorption sites [31]. In contrast, a reduction in stiffness was the case for all tested materials above 20 °C and 40% RH. The finding is in agreement with the adsorption of water by the composite, which can cause the wood to swell, leading to a reduction of the interfacial strength and consequently a decrease in the composite strength [34]. The composites with higher content of wood particles and bigger particles also absorb more water, which could further weaken the bond between polymer (non-polar) and wood (hydrophilic) [33,34].

### 3.4. Shape Change of Bimaterial Actuators in Step-Change Climate

The change in the shape of bilayer actuators depends on selected material properties (i.e., differential expansion), mechanical stiffness, and thickness ratio of materials in the composite [35]. Building on Timoshenko’s theory for hygroscopic bilayers, the thickness of the active layer (t_a_) should be greater than the thickness of the constrained (i.e., passive) layer (t_p_) for both the woven pattern and the simple bonding model [36]. According to the theory, the highest degree of bending is achieved for the ratio of thicknesses *m* = t_p_/t_a_ = √*n*/*n*, considering the ratio of moduli (*n*) of the passive (MOE_p_) and active (MOE_a_) layer in the composite (*n* = MOE_p_/MOE_a_).

(9) $$\frac{1}{R_{T}}-\frac{1}{R_{T_{0}}}=\frac{6\left(\alpha_{a}-\alpha_{p}\right){\left(1+m\right)}^{2}}{3{\left(1+m\right)}^{2}+\left(1+m\cdot n\right)\left(m^{2}+\frac{1}{m\cdot n}\right)}\times \frac{T-T_{0}}{s}$$

where

*R_T_* = radius at temperature *T* (or EMC),
*R*_*T*0_ = radius at temperature *T*_0_ (or initial EMC),
*α* = thermal/hygro expansion coefficients of the materials, where material 1 is the passive component and material 2 is the active component), and
*s* = total thickness of the bilayer material (t_p_ + t_a_).

**Figure 4:**
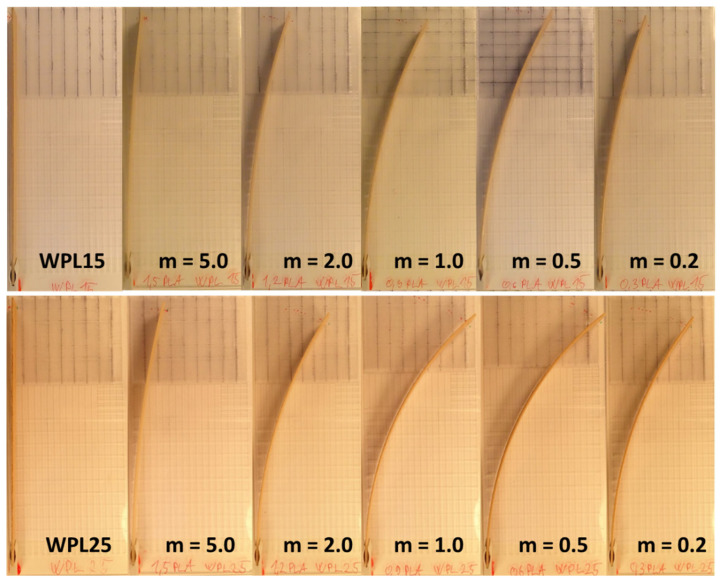
Curvature of WPL15 (**above**) and WPL25 (**bottom**) specimens at the end of the humidification process (168 h at 80% RH and 20 °C) First specimen in a row is pure WPL; from left the thickness of passive (PLA) to active (WPL) decreases from 5.0 to 0.2.

When we take into consideration the calculated moduli of elasticity of source polymers in the initial (i.e., dry) climate (Table 2), and the thickness (s) of 1.8 mm of the bilayered composite, the highest deflection of the composite at the end of humidification should occur with the thickness of the passive layer (PLA) between 0.82 (WPL15) and 0.84 mm (WPL25). The deflection of the bilayered specimens at the end of the humidification process of both tested WPL materials (WPL15, WPL25) showed that the highest bending occurred for actuators with a thickness of PLA of 0.6 and 0.9 mm (0.5 < m < 1.0) (Figure 4). The results are in the range of calculation and follow the theory specified in [35].

**Figure 5:**
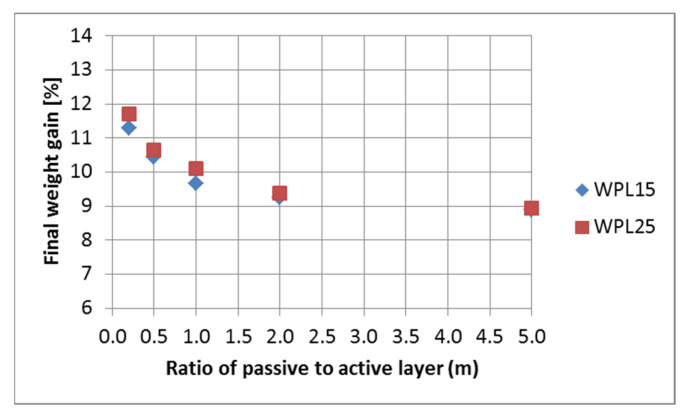
Final weight gain of the bilayered composite in relation to the thickness of the passive (PLA) to active (WPL) layer.

The maximum deflection of the tested bilayered composites is positively correlated with their weight gain during the humidification process (Figure 5). Moisture adsorption was expected to be the highest for composites with the lowest passive layer content. Higher values were confirmed for two-layer composites with a higher proportion of wood fibers (WPL25). The results show that the reduction of the thickness of the constrained layer (PLA), in conjunction with the presumed increase in porosity [24] of the hygroscopic layer (WPL15 or WPL25), is an effective strategy to reduce the negative effects of high bending stiffness on actuation while maximizing moisture exchange and thus responsiveness.

Simple cubic specimens were used to determine the deflection of two-layer actuators. This type of specimen could also be made using a mold casting process, but mold cast materials behave differently due to the pressure applied during fabrication and thus higher density. The 3D printing of simple shape samples was used to obtain basic properties of the 3D printed materials to be used for further studies on more complex structures. Using existing equipment or a 3D printer with dual extruders and modified slicing software, it is possible to print complex structures with different materials at selected positions [37].

### 3.5. Dynamics of Hygromorphism and Moisture Sorption of Bimaterial Actuators

**Figure 6:**
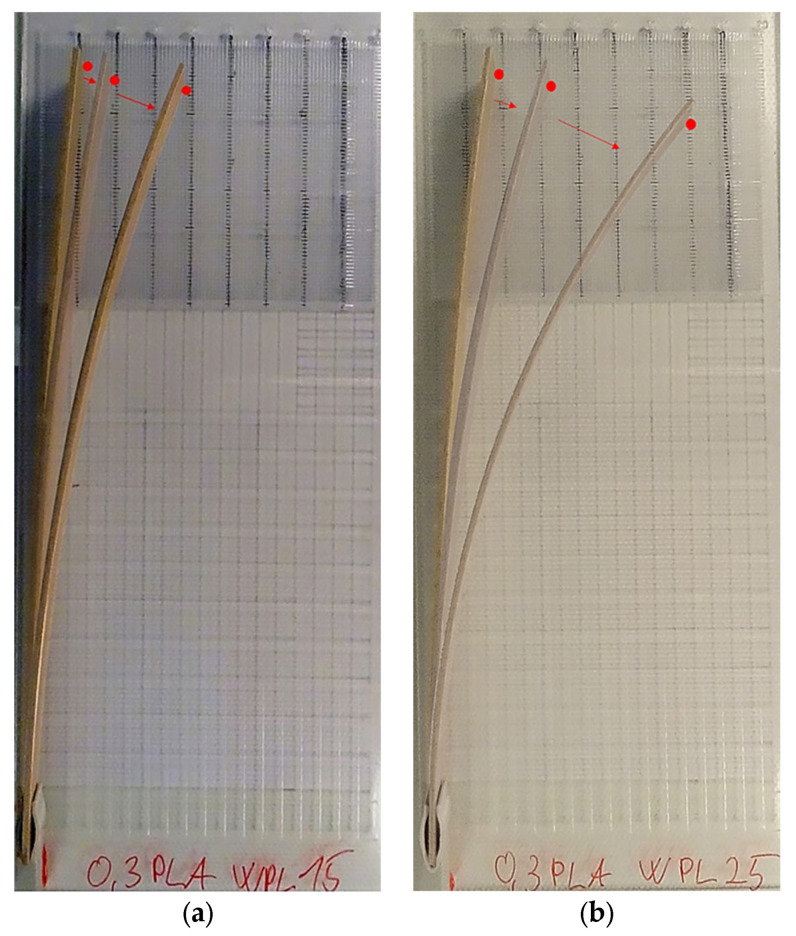
Deflection of the sample 0.3PLA WPL15 (m = 0.2) (**a**) and sample 0.3PLA WPL25 (m = 0.2) (**b**) from the initial position to 6 h (middle red dot) and at the end of humidification process (right red dot) (168 h at humid climate of 80% RH and 20 °C).

**Figure 7:**
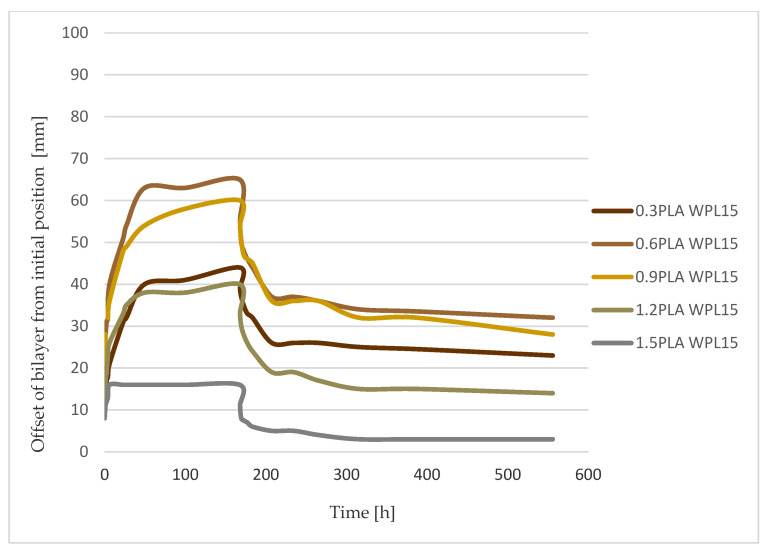
Curvature/offset from initial position of WPL15 bilayer actuators during humidification (up to 168 h) and drying cycle.

**Figure 8:**
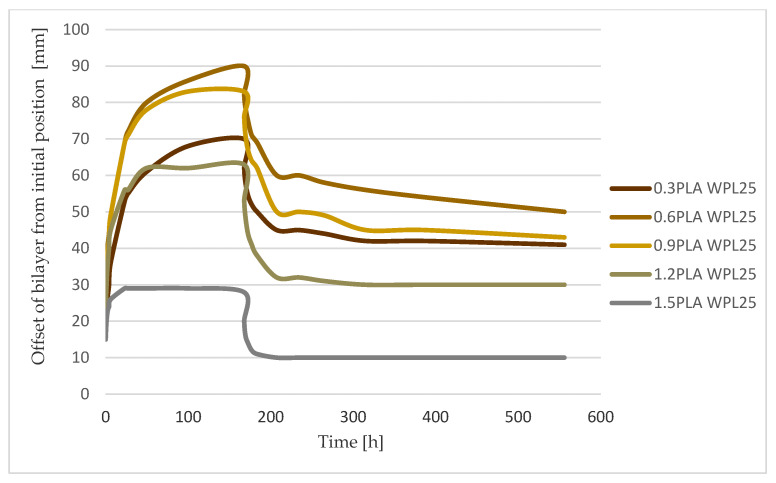
Curvature/offset from initial position of WPL25 bilayer actuators during humidification (up to 168 h) and drying cycle.

To estimate the dynamics of the hygromorphism of bilayered composite to reach equilibrium in a step change of surrounding relative humidity, an FOS with established time constants, equal to the adsorption process, has proven to be effective. The shape change, determined by measuring endpoint position and curvature building during the transient time of tested bilayered composites, followed the theory in every tested case (Figure 6, Figure 7 and Figure 8).

**Figure 9:**
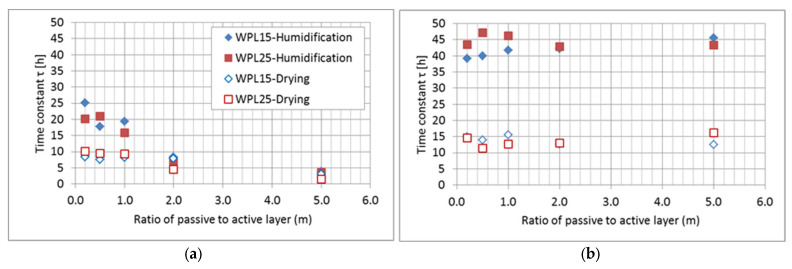
The rate of change of the deflection (**a**) and mass (**b**) of tested bimaterial actuators, determined by the time constant, in relation to the ratio of the thickness of the passive (PLA) to the active layer (WPL).

The obtained response rate in changing the shape of the tested bilayered composites, both WPL15 and WPL25, was practically inversely proportional to their obtained deflection amplitude. In fact, the fastest response was observed in the specimen that had a high ratio between the thickness of the passive to the active layers (m ≥ 2) but achieved only low final deflections. This was confirmed both in humidification and in drying the specimens (Table 3; Figure 6 and Figure 9). Much slower dynamics of the hygromorphism of the samples was confirmed in the samples that had a higher percentage of active layer (m < 1), especially in the humidification process. In the composition, when the samples reach the maximum final amplitude of deflection (0.82 < m < 0.84), the rate of change is up to three times slower when they are moistened and twice as slow when they are dried.

If we compare the dynamics of the hygromorphism of bimaterial actuators with the dynamics of their wetting, we find that they differ (Table 3; Figure 9). In the dynamics of the mass changes of the samples, we could not confirm the characteristic influence of the thickness ratio (m) of the passive to the active layer in the bimaterial composite. This is true for both wetting and drying of the specimens, with the latter process being about three times slower. It is also important to note that the dynamics of moisture adsorption is slower, and the dynamics of drying is approximately the same as the dynamics of the hygromorphism of the tested bimaterial actuators.

It was presumed that specimens with higher wood content (WPL25), due to greater moisture adsorbance capacity (Figure 5), would take longer to reach mass equilibrium. This study did not confirm this. We believe that this is partly a consequence of the higher diffusion coefficient for moisture transport, found for WPL, with higher wood content (Figure 3). Actuation potential is claimed to be directly controlled by fiber microstructure (cellulose microfibril angle and lumen size) and biochemical composition (pectins, hemicelluloses, and lignin) [36]. The authors also claim that the reactivity of the composites is directly linked to the rate of water sorption and transport in wood. This study, in relation to the dynamics of the hygromorphism of the tested bimaterial composites, cannot fully support the earlier findings.

The increase in wood fiber content also leads to higher porosity [24]. 3D-printed biocomposites could have a microstructure with relatively high porosity (even around 20%), leading to damage mechanisms but also to high and rapid water absorption and swelling [18]. Printing parameters (i.e., temperature, speed) could also be optimized for various formulations to influence the performance of an actuator. Hygroscopic stresses are the driving force behind the reactivity of hygromorphic materials and must therefore be maximized. However, it is important to note that a higher percentage of wood in the filament is often reflected in higher dimensional swelling, but also lower tensile strength, a rougher surface, more voids in the material, and visible clusters of wood particles (due to the accumulation of wood particles, causing clogs in the printer nozzle) [24,33]. Filaments with 15 and 25% were used in this study, based on previous research into the printability of wood-PLA. Filaments with high fiber content and consequently higher melting viscosity are typically difficult to handle with the FDM devices [18]. Since we have confirmed slower sorption than the process of hygromorphism of bimaterial actuators in this study, a moisture distribution profile and the associated dimensional changes and stress distribution in the cross-section of the samples emerge. Therefore, for a better understanding of the hygromorphism of the tested composites, it would be useful to investigate the evolution of the moisture and stress field in the cross-section of the test specimens in the future. The changing of stiffness of source polymers in relation to the moisture content, as found in this study, should also be further studied. Nevertheless, in future studies, it would be of great interest to use computed tomography and microscopy.

The maximum deflection of a bimaterial composite (Figure 7 and Figure 8) has linear dependence on the weight gain of an individual bilayer. Both parameters, maximum curvature and weight gain of the sample, are unquestionably related to the hygroscopicity of wood, defined as the ability to exchange moisture with the surrounding environment through processes of adsorption and desorption [38]. Nevertheless, there is no clear answer to the question of optimal deflection behavior of the tested composites. Since the dynamics of hygromorphism are faster in composites with a lower percentage of active layers, a compromise between the two quantities must be found when using bimaterial actuators.

## 4. Conclusions

In summary, bimaterial actuators made from PLA and wood-PLA composites show the potential to be used as hygro-induced shape-changing products. The addition of wood fibers does not influence the differential swelling, although it does affect hygroscopicity.

This study confirms the importance of analyzing the different hygromechanical values of bimaterial composites to understand and predict their hygromorphism after being exposed to different air climates. Strains and mass change are greater for bimaterial composites with higher wood content (WPL 25), while diffusivity for moisture transport is also greater for the latter. The dynamics of the hygromorphism of bimaterial composites and their responsiveness is greater compared to the moisture sorption and when the ratio of the active to the passive layer is low. The opposite is true for the maximum amplitude of composite deflection; the optimal ratio seems to be bilayers with five layers of wood-PLA and one layer of pure PLA (specimens labelled as 0.3PLA WPL), namely specimens with an active/passive ratio (m-ratio) of 5:1. However, the repeatability, accuracy, and optimization of printing parameters and parameters in filament production are essential.

Research has shown that PLA and wood-PLA materials can be used for 3D-printed shape-changing actuators that change in alternating climate conditions. Further research is still needed to evaluate moisture and stress distribution in the tested products as well as long-term behavior in potential applications.

## Figures and Tables

**Table 1:** Mean weight gain, length expansion, and differential swelling (length expansion per % of moisture change) of tested specimens after humidification cycle.

Specimen	Final Weight Gain (%)	Length Expansion (%)	Differential Swelling (%/%)
PT	0.61	0.09	0.233
WPL 15	1.91	0.30	0.226
WPL 25	2.82	0.47	0.222

**Table 2:** Mean modulus of elasticity (MOE) and moisture content (MC) for specimens conditioned at 20 °C between 20% and 80% RH.

T = 20 °C	PLA			WPL 15			WPL 25		
RH (%)	MOE (GPa)	St. Dev.	MC (%)	MOE (GPa)	St. Dev.	MC (%)	MOE (GPa)	St. Dev.	MC (%)
20	2.91	0.168	0.10	2.16	0.115	0.43	2.32	0.206	0.59
40	3.30	0.149	0.31	2.36	0.118	1.30	2.50	0.252	1.86
60	3.11	0.222	0.44	2.17	0.139	1.56	2.24	0.237	2.27
80	2.83	0.162	0.62	1.97	0.096	2.31	2.00	0.218	3.41

**Table 3:** Dynamics of weight change and shape change of tested biomaterial determined by time constant τ (tau) in First Order system (tp—thickness of passive layer, ta—thickness of active layer, m—ratio of thicknesses of active to passive layer).

Specimen	PLA	WPL	Ratio	Humidification			Drying	
				Mass		Deflection	Mass	Deflection
	t_p_ (mm)	t_a_ (mm)	m	Final Weight Gain (%)	τ (h)	τ (h)	τ (h)	τ (h)
0.3 PLA WPL15	0.3	1.5	0.2	11.3	39.2	25.1	14.9	8.4
0.6 PLA WPL15	0.6	1.2	0.5	10.4	40.0	17.7	13.9	7.5
0.9 PLA WPL15	0.9	0.9	1.0	9.7	41.8	19.3	15.6	11.2
1.2 PLA WPL15	1.2	0.6	2.0	9.3	42.2	8.4	12.6	7.8
1.5 PLA WPL15	1.5	0.3	5.0	8.9	45.5	3.8	12.5	3.0
WPL15	0.0	1.0	0.0	10.9	38.5	2.1	25.0	6.6
0.3 PLA WPL25	0.3	1.5	0.2	11.7	43.5	20.1	14.5	10.1
0.6 PLA WPL25	0.6	1.2	0.5	10.6	47.1	20.9	11.4	9.5
0.9 PLA WPL25	0.9	0.9	1.0	10.1	46.2	15.9	12.7	9.3
1.2 PLA WPL25	1.2	0.6	2.0	9.4	42.9	7.0	12.9	4.5
1.5 PLA WPL25	1.5	0.3	5.0	8.9	43.3	3.5	16.2	1.4
WPL25	0.0	1.0	0.0	11.8	24.4	2.5	10.1	1.6
PLA	1	0		7.2				

## Data Availability

The data presented in this study are available on request from the corresponding author.
